# Proteomic analysis of early salt stress responsive proteins in alfalfa roots and shoots

**DOI:** 10.1186/s12953-017-0127-z

**Published:** 2017-10-30

**Authors:** Junbo Xiong, Yan Sun, Qingchuan Yang, Hong Tian, Heshan Zhang, Yang Liu, Mingxin Chen

**Affiliations:** 1 Hubei Key Laboratory of Animal Embryo and Molecular Breeding, Institute of Animal and Veterinary Science, Hubei Academy of Agricultural Science, Yaoyuan 1, Hongshan, Wuhan, Hubei 430017 China; 2 Institute of Grassland Science, China Agricultural University, 2 West Road, Yuan Ming Yuan, Beijing, 100193 China; 3 Institute of Animal Science, Chinese Academy of Agricultural Science, West Road 2, Yuan Ming Yuan, Beijing, 100193 China

**Keywords:** NaCl stress, *Medicago sativa* root and shoot, Two-dimensional electrophoresis, Differentially abundant proteins

## Abstract

**Background:**

Alfalfa (*Medicago sativa*) is the most extensively cultivated forage legume in the world, and salinity stress is the most problematic environmental factors limiting alfalfa production. To evaluate alfalfa tissue variations in response to salt stress, comparative physiological and proteomic analyses were made of salt responses in the roots and shoots of the alfalfa.

**Method:**

A two-dimensional gel electrophoresis (2-DE)-based proteomic technique was employed to identify the differentially abundant proteins (DAPs) from salt-treated alfalfa roots and shoots of the salt tolerance cultivars Zhongmu No 1 cultivar, which was subjected to a range of salt stress concentrations for 9 days. In parallel, REL, MAD and H_2_O_2_ contents, and the activities of antioxidant enzymes of shoots and roots were determinand.

**Result:**

Twenty-seven spots in the shoots and 36 spots in the roots that exhibited showed significant abundance variations were identified by MALDI-TOF-TOF MS. These DAPs are mainly involved in the biological processes of photosynthesis, stress and defense, carbohydrate and energy metabolism, second metabolism, protein metabolism, transcriptional regulation, cell wall and cytoskeleton metabolism, ion transpor, signal transduction. In parallel, physiological data were correlated well with our proteomic results. It is worth emphasizing that some novel salt-responsive proteins were identified, such as CP12, pathogenesis-related protein 2, harvest-induced protein, isoliquiritigenin 2′-O-methyltransferase. qRT-PCR was used to study the gene expression levels of the four above-mentioned proteins; four patterns are consistent with those of induced protein.

**Conclusion:**

The primary mechanisms underlying the ability of alfalfa seedlings to tolerate salt stress were photosynthesis, detoxifying and antioxidant, secondary metabolism, and ion transport. And it also suggests that the different tissues responded to salt-stress in different ways.

## Background

Soil salinity is a world-wide problem, but is most acute in North and Central Asia, South America, Australasia, and the Mediterranean area. The soil solution in saline soils is composed of a range of dissolved salts, such as NaCl, Na_2_SO_4_, MgSO_4_, CaSO_4_, MgCl_2_, KCl, and Na_2_CO_3_, each of which contribute to salinity stress. However, NaCl is the most prevalent salt and has been the focus of much of the work on salinity to date [1, 2]. High NaCl concentrations affect plant physiology and metabolism at different levels. High concentrations can cause water deficits, ion toxicity, nutrient imbalance, and oxidative stress, leading to molecular damage, growth and yield reductions, and even plant death.

Alfalfa (*Medicago sativa* L.) is a perennial warm-season forage legume with a high yield and good nutrient contents (crude protein content can reach approximately 16% to 22%), and can be grown on more than 30 Mha worldwide. However, its yield is low in arid and semi-arid regions where salinity is the main problem. Alfalfa is moderately tolerant to salinity when the electrical conductivity (EC) is 2.0 dS/m (1280 ppm) and the soil osmotic potential threshold is 1.5 bars (1 bar = 0.987 atm) at field capacity. An additional 7% decrease in alfalfa yields can be expected with each dS/m increase in saturation extract salinity [3]. Excessive salinity in the crop root zone creates osmotic stress, which reduces root uptake of water and crop transpiration, leading to reduced forage yields [4].

Understanding the alfalfa tolerance mechanisms to high concentrations of NaCl in soils may ultimately help to improve yields on saline lands. Previous studies indicated that alfalfa salt tolerance is generally associated with modifications of morphological and physiological traits, such as changes in plant architecture and growth (shoots and roots), variations in leaf cuticle thickness, stomatal regulation, germination, and photosynthesis rate. These changes are linked to diverse cellular modifications, including, changes in membrane and protein stability, increased antioxidant capacity and activation of hormonal signaling pathways, notably those depending on the “stress hormone” abscissic acid [5]. The regulation of these changes at the cellular level are the main responses that cause alterations in gene expression and several attempts have been made to obtain a profile for gene expression in alfalfa under saline conditions [6, 7]. However, transcript profiles do not always provide a complete story due to limited correlations between the transcript and protein levels, and proteomics has become a critical complement to mRNA data and an improved biological view of plant biology. Currently, several studies have attempted to analyze alterations in protein expression in response to salt, and proteomics studies that focused on 34 plant species have identified 2171 salt-responsive protein identities, representing 561 unique proteins [8]. To date, few studies have investigated the effects of salt stress on alfalfa.

Salt stress induces many different proteomic changes in various plant tissues due to their distinct functions and growth environments. A comparative analysis of different plant tissue responses to salinity stress at the same time would improve understanding of different tissues protein compositions and their differential responses to salinity stress. Furthermore, it would provide further insights into the proteomic mechanisms controlling salt tolerance. A few previous studies examined protein change responses in different tissues to salinity stress, such as the report on soybean (*Glycine max* L.) leaves, hypocotyls, and roots [9, 10], creeping bentgrass (*Agrostis. stolonifera* L.) leaves and roots [11], and rice (*Oryza sativa* L.) leaves and roots [12]. They all suggested that protein responses to salt-stress in different tissues varied and some protein showed tissue specific abundance.

Alfalfa cultivar “Zhongmu No1”, one salt tolerance cultivar commonly used in China agriculture, was released by the Chinese Academy of Agricultural Science in 2001. This germplasm represents the four cycle of recurrent mass selection for alfalfa genotypes that germinate at high levels of NaCl. In this study, we analyzed the “Zhongmu No1” cultivar shoot and root responses to different NaCl concentrations using physiological and biochemical methods, and comparative proteomics. Based on our findings, we produced a possible schematic representation of the mechanism associated with salt tolerance in alfalfa.

## Methods

### Plant materials and stress treatments

Alfalfa seeds (*Medicago sativa* L.cv. Zhongmu No 1) were germinated in the dark for 48 h at 28 °C, then transplanted into 1/2 Hoagland’s nutrient solution and grown on for 7 days. Subsequently, the seedlings were subjected to 0 (control), 100, and 200 mM NaCl 1/2 Hoagland’s nutrient solution for 9 d. The salt concentration was maintained by a daily input of 50 mM NaCl. The experiments were conducted in a glasshouse chamber that had an average temperature of 27 °C/18 °C day/night, and a light irradiance of 150 μmol m^−2^ s^−1^.

### H_2_O_2_, MDA, and relative electrolyte leakage analyses

For the H_2_O_2_ content analysis, 1 g each of root and shoot tissues were ground in liquid N_2_ and then homogenized in 5 ml cold acetone. The supernatants were used for H_2_O_2_ content assays after centrifugation at 3000 *g* and 4 °C for 10 min. The H_2_O_2_ content was assayed by analyzing the production of titanium–hydroperoxide complex at 410 nm [13]. MDA was measured using a modified thiobarbituric acid (TBA) method as described previously [14]. Relative electrolyte leakage was determined by modifying a method described previously [15]. A total of 500 mg of tissues were rinsed with ddH_2_O, placed in test tubes containing 10 ml of ddH_2_O, and incubated at room temperature for 2 h. The electrical conductivity of the solution (C_1_) was measured using a conductivity meter (DDS-307A; China). Then the tubes were boiled for 15 min, cooled to room temperature, and the electrical conductivity (C_2_) measured again. The REL was calculated by the formula: C_1_ / C_2_ × 100%.

### SOD, APX, POD, and CAT activity analyses

The enzyme extraction and enzyme activity assays were determined by methods modified from those previously described [16]. Root and shoot samples (200 mg each) were ground into fine powder with liquid nitrogen in a pre-chilled mortar and pestle. Further grinding was performed in a solution of 50 mM potassium phosphate buffer pH 7.0 containing 1 mM EDTA and 2% (*w*/*v*) polyvinylpolypyrrolidone (PVPP) for the APX and CAT assays, and in a solution of 50 mM potassium phosphate buffer at pH 7.0 containing 0.5 mM EDTA for the SOD and POD assays. The homogenates were centrifuged at 14000 *g* for 15 min at 4 °C. The resulting supernatants were centrifuged again and used immediately for enzyme activity assays or stored at −30 °C to be used later. Total SOD (EC 1.15.1.1) activity was determined by monitoring its ability to inhibit the photochemical reduction of nitro blue tetrazolium (NBT). APX activity (EC 1.11.1.11) was determined by following the decrease in ascorbate and measuring the change in absorbance at 290 nm over 2 min intervals.The POD (EC 1.11.1.7) and CAT (EC 1.11.1.6) activity were determined by following the decrease in H_2_O_2_, and measuring the change in absorbance at 240 nm over 2 min intervals.

### Protein extraction and 2-DE

The total proteins were extracted by a modified TRIzol reagent method, which was recently developed to obtain high-quality proteins from *Medicago truncatula* tissues for 2-DE [17]. The whole roots and shoots were cut off the seedlings, frozen in liquid nitrogen and ground to a fine powder for protein extraction. Finally, the pellets were dried in a freeze-vacuum dryer for 10 min, resuspended in 1.5 mL lysis buffer (8 M urea, 4% *v*/v CHAPS, 2% *w*/*v* DTT), sonicated (10 min) at 4 °C and incubated at room temperature for 2 h. The supernatant was collected after centrifugation (40 min, 40,000 *g*, 4 °C). The protein concentration of the supernatant was determined using a 2-D Quant kit, following the manufacturer’s protocol.

Samples containing 120 μg total protein in 450 μL rehydration buffer (8 M urea, 2% w/v CHAPS, 1% w/v DTT, 0.5% v/v IPG buffer pH 4–7, 0.002% w/v bromophenolblue) were loaded onto a 24 cm, pH 4 to 7 linear gradient IPGstrip (GE Healthcare, USA). IEF was carried out using an Ettan IPGphorII (GE Healthcare, Uppsala, Sweden). Focusing was performed at 20 °C as follows: active rehydration at 30 V for 12 h, 150 V for 1 h, 500 V for 1 h, 1000 V for 1 h, 8000 V for 2 h, and 8000 V up to 40,000 VH. After IEF, the proteins were equilibrated as described. First the IPG strips were incubated in 10 mL of equilibration buffer (6 M urea, 30% *w*/*v* glycerol, 2% w/v SDS, 50 mM Tris-HCl, pH 8.8) with 1% w/v DTT for 15 min, and then in the same solution containing 2.5% w/v iodoacetamide instead of DTT for 15 min. Following this, the strips were transferred to 12% SDS-PAGE gels for second dimension electrophoresis with the Ettan DALTsix gel system (GE Healthcare, Uppsala, Sweden), using SDS electrophoresis buffer (250 mM Tris-base, 1.92 M glycine, 1% w/v SDS) with a 0.2 W/strip for 1 h, and a 15 W/strip until the dye front reached the bottom of the gel. All 2-DE separations were repeated three times for each tissue extract.

### Protein visualization, image analysis

Upon electrophoresis, Gels gels were stained with silver nitrate according to GE handbook (GE Healthcare, Uppsala, Sweden) with some modifications. Briefly, gels were fixed in 40% ethanol and 10% acetic acid for 60 min, and then sensitized with 30% ethanol, 0.2% sodium thiosulfate *w*/*v*, and 6.8% sodium acetate w/v for 30 min. Then gels were rinsed with distilled water three times, 5 min for each time, then incubated in silver nitrate (2.5 g/L) for 20 min. Incubated gels were rinsed with distilled water two times, and developed in a solution of sodium carbonate (25 g/L) with formaldehyde (37%, w/v) added (240 mL/L) for two times, first for 1 min, then stained for 4 min. Development was stopped with 1.46%w/v Ethylene Diamine Tetraacetic Acid for 10 min, then gels were rinsed with distilled water three times, 5 min for each time. Gels were stored in distilled water until they could be processed.Gels images were acquired using a PowerLook 2100XL color scanner (UMAX Technologies, CA, USA) and analyzed with Image master 2D Platinum Software Version 6.0 (GE Healthcare, Uppsala, Sweden).

### Protein identification by MALDI-TOF-MS/MS

Proteins were identified by MALDI-TOF-MS/MS. Selected spots were excised from the gels and destained with a solution containing 20% *w*/*v* sodium thiosulphate and 1% w/v potassium ferricyanide for 5 min. The supernatant was removed and the gel spots were washed twice with 25 mM ammonium bicarbonate in 50% *v*/v acetonitrile for 20 min. The gel spots were then washed in acetonitrile, dried in a Speed-Vac and digested overnight with 20 μg/mL trypsin in 25 mM ammonium bicarbonate at 37 °C. Tryptic peptides were passed through C18 Zip-Tips and mixed with 5 mg/mL of R-cyano-4-hydroxycinnamic acid, as the matrix, and subject to MALDI-TOF/TOF analysis (4700 Proteomics Analyzer, Applied Biosystems). Data files obtained from MALDI-TOF/TOF mass spectra were submitted to the Mascot search engine using Daemon 2.1.0 (Matrix Science; http://www.matrixscience.com) on Mascot server version 2.2.1. The data were searched against the NCBInr database and the peptides were constrained to being tryptic with a maximum of one missed cleavage. Carbamidomethylation of cysteine was considered a fixed modification, and oxidation of methionine residues was considered as a variable modification. The identification was based on the combination of a high Mascot score and maximum peptide coverage.

### qRT-PCR analysis

Total RNA was extracted from salt-treated and control alfalfa roots and shoot by Trizol reagent (TaKaRa), and cDNA was reverse transcribed from 1 μg of to total RNA using a First Strand cDNA Synthesis Kit (Invitrogen). Gene-specific primers (GSPs) used for qRT-PCR were designed using primer 5 according to cDNA sequences obtained from the alfalfa (Table 1). The alfalfa Actin gene was used as an endogenous control for normalization. The PCR reaction was carried out in a 20 uL volume containing 10 μL 2 × SYBR Green Master Mix reagent (TaKaRa), 1 μL template cDNA and 0.5 μL of each GSPs with the following reaction conditions: 95 °C for 30 s; followed by 40 cycles of 95 °C for 10 s; 55 °C for 10 s and 72 °C for 15 s. Relative gene expression was calculated using the ddCt alogorithm [18].

**Table 1:** The primers for qRT-PCR

Protein	Genes	Primers	Sequence
Actin	gi|378407816	Forward primer(5′-3′)	GATACTCTTTCACCACAACAGCCG
		Reverse primer(5′-3′)	ACTTCAGGACAACGGAAACGCT
CP12	gi|3,123,345	Forward primer(5′-3′)	TGGCAACAATAGGTGGTCT
		Reverse primer(5′-3′)	CTCGTCGGTTTCAGGGT
HI protein	gi|283,831,548	Forward primer(5′-3′)	GCTGATGAAATCGTCCCA
		Reverse primer(5′-3′)	ACCCTGTTCCTCCCACTAAGCTGTA
PR protein 2	gi|44,887,779	Forward primer(5′-3′)	CTAAATTACCAGCATCAACGC
		Reverse primer(5′-3′)	CCTCTACTTTCATCAGGGACAA
IOMT	gi|22,266,001	Forward primer(5′-3′)	GCTGATGAAATCGTCCCA
		Reverse primer(5′-3′)	AACCCTGTTCCTCCTACCA

### Immunoblot analysis

Protein samples (50 mg/lane) were separated using 12% one dimensional SDS-PAGE gel electrophoresis, transferred onto nitrocellulose membranes, and incubated at room temperature for 2 h with rabbit polyclonal antibodies raised against Rubisco activase, Heat shock protein 70 each (Agrisera, Sweden) at 1:5000 dilution. After washing three times with TBST buffer (0.01 M TBS, 0.1% Tween-20, pH 7.6), the membranes were exposed for 2 h at room temperature to horseradish peroxidase-conjugated goat anti-rabbit IgG at 1:300 dilution. Positive signals were visualized with 3, 3′-diaminobenzidine.

### Statistical analysis

Data from repeated measurements are shown as mean. Comparison of differences among the groups was carried out using Student’s test. Significant differences were determined relative to the *P* value [*P*-values <0.05 (*) and <0.01 (**)].

## Results

### Changes in REL and MAD contents

**Fig. 1:**
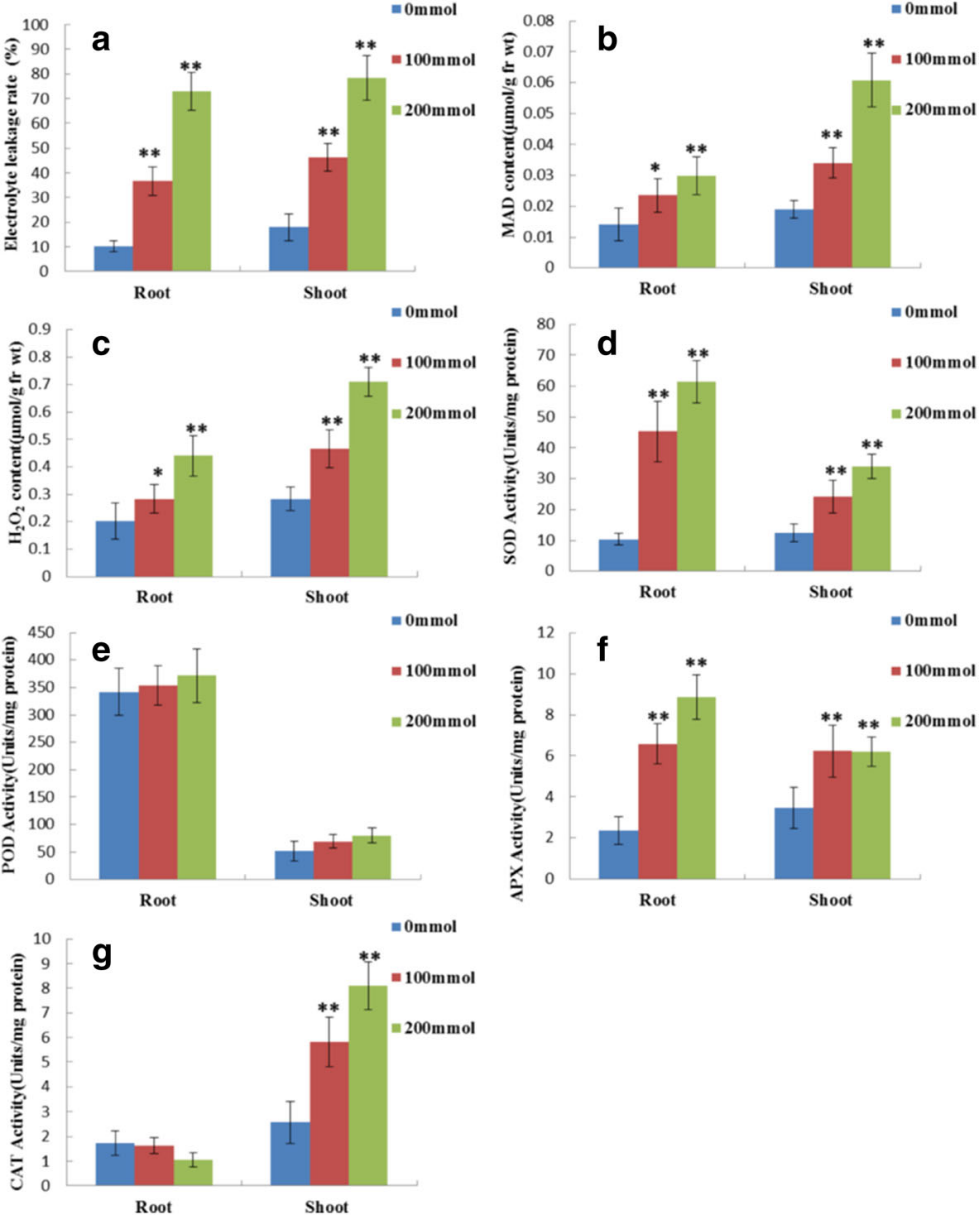
Physiological responses induced by NaCl treatment (0,100, 200 Mm) for 9 days in *Medicago sativa* leaves and roots. Effects of salinity on the Relative electrolyte leakage (**a**), MAD content (**b**), H_2_O_2_ content (**c**), SOD activity (**d**), POD activity (**e**), APX activity (**f**), CTA activity (**g**) were presented. Significant differences were determined relative to each treatment using a student’s t-test [*P*-values <0.05 (*) and < 0.01 (**)]. Bars: SD

REL and MAD are indicators of membrane damage caused by NaCl stress. Stress-induced REL and MAD changes in the roots and shoots are shown in Fig. 1a, b. These data demonstrated a significant increase in the REL and MAD (*P*-values <0.05 and P-values <0.01) when alfalfa seedlings were treated with 100 mM and 200 mM NaCl. The roots had higher REL and MAD contents than the shoots.

### Changes in H_2_O_2_ and antioxidant enzyme activities

As shown in Fig. 1c, a significant increase in the H_2_O_2_ when alfalfa seedlings were treated with 100 mM and 200 mM NaCl (*P*-values <0.05 and *P*-values <0.01). The shoots had higher H_2_O_2_ contents than the roots. Under normal conditions, the SOD activity was higher in the shoots than in the roots, and it was significant increase (*P*-values <0.01) in roots and shoots when alfalfa seedlings were treated with 100 mM and 200 mM NaCl (Fig. 1d). The SOD in the roots was 3.78 and 5.29 times higher in 100 and 200 mM NaCl, respectively, than in the control, and was 1.59 and 2.35 times higher than in the shoots. Similarly, the APX activity was significant increase (*P*-values <0.01) in the shoots and roots as the NaCl concentration increased. Furthermore, the rate of increase in APX activity in the shoots was slower than in the roots (Fig. 1f). Salinity effects on POD activity are shown in Fig. 1e. Under normal conditions, the POD activity in the roots was 5.48 times higher than in the shoots. Salt stress slightly increased the POD activity in the roots and shoots, but it was not significant (*P*-values >0.05). The salt stress treatments up–regulated CAT activity by 2.15 and 2.91 times respectively, in shoot. However, the CAT activity in the roots slightly decreased under salt stress, it was not significant (*P*-values >0.05) (Fig. 1g).

### Identification and functional classification of DAPs

**Fig. 2:**
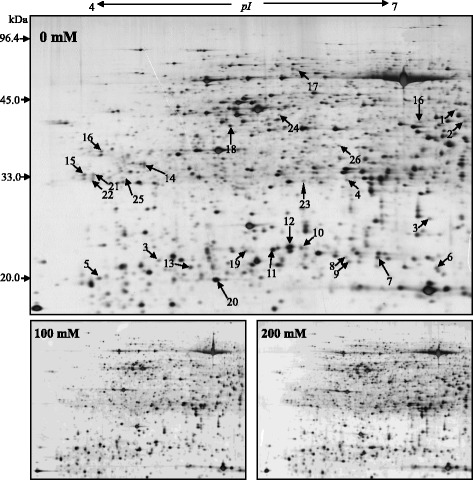
2-DE analysis of proteins extracted from alfalfa shoot under different salinity. Arrows indicate protein changes induced by NaCl treatment

**Fig. 3:**
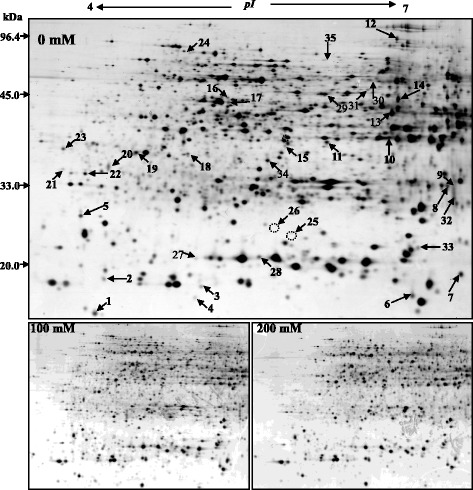
2-DE analysis of proteins extracted from alfalfa roots under different salinity. Arrows indicate protein changes induced by NaCl treatment

**Fig. 4:**
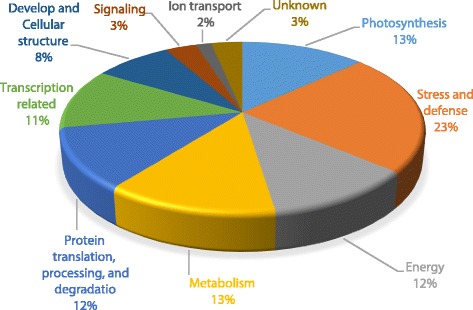
Functional classification of differentially abundant proteins identified in the seedling shoot and root of alfalfa under salt stress. The pie chart shows the distribution of the salt-responsive proteins into their functional classes in percentage

More than 850 proteins were detected in each gel by ImageMaster software (Figs. 2 and 3). Comparison of control and salt-treated plants reference gels allowed the identification of differentially spots. Differentially spots were selected based on the following criteria: (i) relative vol% of the spot with fold change in a comparison >1.5 or <0.67; (ii) unadjusted significance level *p* < 0.05. Then the spots were analyzed by MALDI-TOF-TOF MS, and a total 61 DAPs were identified: 26 spots in the shoots and 35 spots in the roots (Table 2). Differentially expressed proteins were classified based on KEGG (http://www.kegg.jp/kegg/pathway.html) and previous literature (Fig. 4). In the shoots, the largest two groups were photosynthesis (31%), and stress and defense (20%) groups. In the roots, the largest three groups were stress and defense (26%), metabolism (17%), and protein translation, processing, and degradation (17%). It is noteworthy that proteins involved in signaling and ion transport were only found in the roots.

**Table 2:** Identification of salt-resposive proteins in alfalfa using MALDI-TOF-MS/MS

Spot No.^a^	Homologous protein (plant species)^b^	gi Number^c^	Theo. Mr./pI^d^	Exp. Mr./pI^e^	Scores^f^	M.P^g^	Relative Vol% ± SE^h^ CK 100 mM 200 mM
Photosynthesis							
2	RuBisCO large subunit [*Medicago sativa*]	1,223,773	12.0/6.1	41.7/6.9	263	3	
3	RuBisCO small subunit [Medicago sativa]	2,342,980	52.3/6.1	27.5/6.7	132	3	
4	RuBisCO large subunit [Medicago sativa]	1,223,773	12.0/6.1	31.5/6.1	99	4	
8	RuBisCO activase [*Oryza sativa* Japonica Group]	1,778,414	24.6/8.6	21.7/6.1	126	5	
12	Cytochrome b6-f complex iron-sulfur [*Pisum sativum*]	136,707	23.1/5.9	23.5/5.7	138	5	
16	Chlorophyll a/b binding protein [*Arabidopsis thaliana*]	16,374	25.0/5.1	36.6/4.5	112	4	
26	Chloroplast oxygen-evolving enhancer protein 1 [Leymus chinensis]	147,945,622	34.5/6.0	35.5/6.1	95	4	
6	CP12 [*Chlamydomonas reinhardtii*]	3,123,345	12.5/5.7	15.3/6.6	92	3	
Stress and defense							
25	Glutathione peroxidase 1 [Lotus japonicus]	37,930,463	26.0/10.0	31.8/4.6	97	7	
23	Chloroplast thylakoid-bound ascorbate peroxidase [*Vigna unguiculata*]	45,268,437	45.1/8.2	31.2/5.8	115	4	
13	Harvest-induced protein [Medicago sativa]	283,831,548	16.6/5.1	21.6/5.1	133	5	
19	Quinone reductase family protein [Arabidopsis thaliana]	30,687,535	21.7/6.1	23.7/5.5	186	6	
20	Pathogenesis-related protein 2 [Medicago sativa]	22,266,001	16.5/5.8	20.1/5.3	344	9	
R4	Glutathione peroxidase [*Medicago truncatula*]	355,524,544	21.3/7.6	11.7/4.6	76	5	
R21	Ascorbate peroxidase [Medicago sativa]	16,304,410	20.1/5.3	34.7/4.3	99	6	
R23	Ascorbate peroxides [Medicago sativa]	16,304,410	20.1/5.3	40.1/4.2	81	5	
R16	Cytosolic ascorbate peroxidase [*Cucumis sativus*]	1,669,585	27.5/5.4	37.9/6.1	102	3	
R9	Pathogenesis-related protein 5 [Arabidopsis thaliana]	15,222,089	26.2/4.8	34.7/6.9	113	5	
R27	Pathogenesis-related protein 2 [Medicago sativa]	22,266,001	16.5/5.8	23.7/5.6	344	9	
R26	Alcohol dehydrogenase-1F [*Phaseolus acutifolius*]	113,361	41.8/6.1	21.3/5.1	101	4	
R10	Alcohol dehydrogenase [*Dianthus caryophyllus*]	33,149,683	41.9/6.6	38.1/6.4	79	3	
R18	Ferritin [*Glycine max*]	968,987	28.0/5.9	34.2/5.1	82	4	
Carbohydrate and energy metabolism							
18	Glyceraldehyde-3-phosphate dehydrogenases [Arabidopsis thaliana]	15,229,231	39.0/6.7	39.5/5.4	117	3	
11	ATP synthase bate subunit [*Kerria japonica*]	7,578,491	48.0/5.5	23.2/5.6	101	5	
R7	Malate dehydrogenase, mitochondrial [Medicago sativa]	32,328,905	48.6/6.5	18.7/6.9	97	3	
R34	Glyceraldehyde 3-phosphate dehydrogenases [Arabidopsis thaliana]	15,229,231	39.0/6.7	22.3/5.6	87	4	
R14	ATP synthase bate china 2 mitochondrial [Arabidopsis thaliana]	18,415,911	73.0/5.8	42.3/6.7	75	4	
R12	Nucleoside diphosphate kinase 1 [Pisum sativum]	134,667	16.4/5.9	95.0/6.5	69	3	
R24	Cytosolic phosphoglycerate kinase [Pisum sativum]	923,077	42.3/5.7	91.2/5.1	522	9	
Second metabolism							
1	Allene oxide cyclase 2 [Arabidopsis thaliana]	18,404,656	38.9/ 5.0	42.7/6.9	91	5	
17	Myo-inositol-3-phosphate synthase [Glycine max]	13,936,691	56.6/5.3	51.3/5.8	87	4	
R5	Isoliquiritigenin 2′-O-methyltransferase [Medicago sativa]	44,887,779	41.5/5.1	25.0/4.3	88	5	
R2	Glutamine synthetase [Arabidopsis thaliana]	28,393,681	44.0/5.15	18.5/4.5	121	5	
R11	3-Isopropylmalate dehydrogenase [Arabidopsis thaliana]	121,343	46.6/6.0	38.0/6.0	104	4	
R13	Chalcone reductase [Medicago sativa]	563,540	35.0/6.5	42.1/6.6	111	6	
R19	Isoflavone reductase [Medicago sativa]	19,620	35.5/5.3	38.5/4.7	129	5	
R33	Chalcone isomerase [Medicago sativa]	166,400	21.4/5.5	22.3/6.6	104	5	
Protein metabolism							
10	Small ribosomal protein 4 [Squamidium brasiliense]	37,992,679	21.9/10.0	23.3/5.8	79	3	
R25	Probable protein disulfide-isomerase A6 [Arabidopsis thaliana]	7,294,421	40.8/5.4	22.3/5.7	544	9	
R32	Ribosomal protein L32 [Medicago sativa]	71,534,997	15.7/10.9	30.2/6.8	69	5	
R29	Mitochondrial processing peptidase [*Solanum tuberosum*]	587,562	54.6/5.9	44.2/6.0	72	4	
R1	Heat shock protein 70 [Cucumis sativus]	1,143,427	75.3/5.1	12.0/4.4	75	4	
R30	Proteasome subunit alpha type-2-B [Arabidopsis thaliana]	15,219,317	25.7/5.5	46.2/6.4	78	4	
R28	Eukaryotic translation initiation factor 5A-2(eIF-5A-2) [*Gossypium barbadense*]	45,644,510	17.2/5.2	21.3/5.6	75	5	
Transcriptional regulation							
7	mRNA binding protein precursor [*Solanum lycopersicum*]	26,453,355	44.0/7.1	21.2/6.3	84	3	
15	Nucleic acid binding protein1 [*Zea mays*]	162,463,757	33.1/4.6	33.1/4.4	102	5	
5	Maturase [*Kopsia fruticosa*]	59,932,902	59.8/9.2	20.0/4.4	88	4	
24	Putative RNA binding protein [*Mesembryanthemum crystallinum*]	388,621	32.0/4.7	43.7/5.7	87	4	
R20	Putative polyprotein [Oryza sativa Japonica Group]	50,300,539	35.6/4.5	35.6/4.5	93	5	
R22	RNA-binding protein [Arabidopsis thaliana]	21,593,201	42.7/7.7	34.2/4.4	85	5	
R35	Nucleic acid binding protein1 [Zea mays]	162,463,757	33.1/4.6	48.8/6.0	89	4	
Cell wall and cytoskeleton metabolism							
21	Cell division cycle protein 48 [Arabidopsis thaliana]	1,841,493	90.6/5.0	33.0/4.5	81	5	
22	Cell division cycle protein 48 [Arabidopsis thaliana]	1,841,493	90.6/5.0	32.8/4.5	73	5	
R6	Actin-depolymerizing factor [Malus x domestica]	33,772,153	11.1/8.7	20.8/6.7	75	4	
R8	Profilin-2 [*Lilium longiflorum*]	14,423,862	14.3/4.6	31.2/6.8	226	8	
R31	Actin-depolymerizing factor [*Malus domestica*]	33,772,153	11.1/8.7	44.9/6.3	107	6	
Signaling							
R17	Annexin [*Lavatera thuringiaca*]	2,459,926	36.2/6.1	44.7/5.3	121	6	
R15	Vacuole-associated annexin VCaB42 [*Nicotiana tabacum*]	4,580,920	36.0/5.3	37.7/5.7	135	5	
Ion transport							
R3	Plasma membrane H^+^- ATPase [*Hordeum vulgare subsp. vulgare*]	15,149,829	70.9/8.6	16.4/5.2	91	4	
Unknown							
9	Unknown protein [Medicago truncatula]	217,071,692	55.5/5.5	21.8/6.1	87	4	
14	Unknown protein [Arabidopsis thaliana]	22,329,503	54.9/5.5	34.2/4.8	145	5	

^a^The number of identification spot

^b^The homologous protein and plant species

^c^The number NCBInr databases

^d^Theoretical mass (*Mr*, kDa) and p*I* of identified proteins. Theoretical values were retrieved from the protein database

^e^Experimental mass (*Mr*, kDa) and p*I* of identified proteins. Experimental values were calculated with Image master software (GE Healthcare) and standard molecular mass markers

^f^The mascot scores

^g^Number of matched petides

^h^The Relative Vol% of spot

### Correlation of 2-DE data with qRT-PCR

**Fig. 5:**
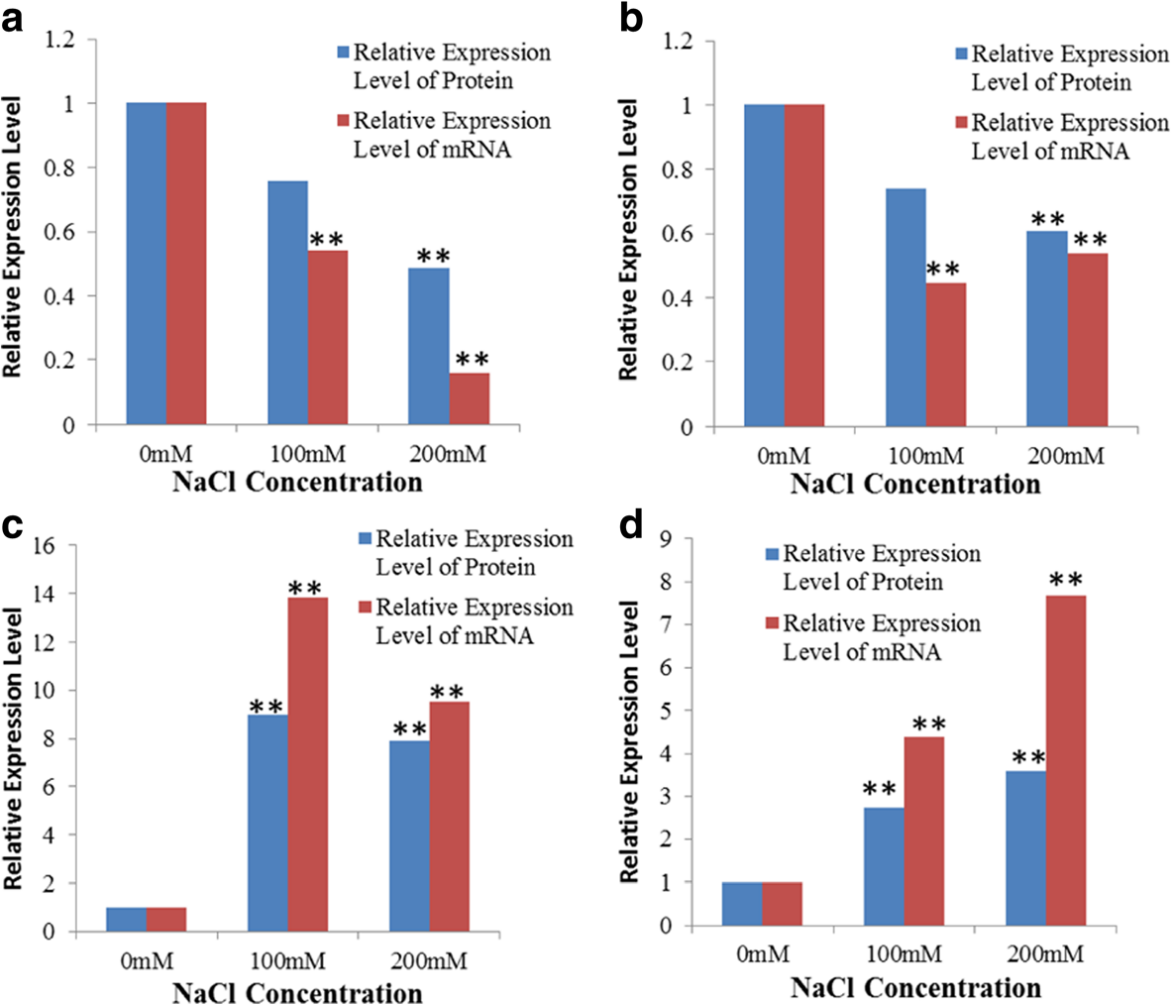
Transcript abundances of mRNAs encoding four novel salt-responsive proteins were analyzed following salt stress treatment. The mRNA levels were compared with the 2-DE data. Significant differences were determined relative to each treatment using a student’s t-test [*P*-values <0.05 (*) and <0.01 (**)]. **a** CP12 (gi|3,123,345). **b** Harvest-induced protein (HI,gi|283,831,548). **c** Pathogenesis-related protein 2 (PR2,gi|44,887,779). **d** Isoliquiritigenin 2′-O-methyltransferase(IOMT, gi|22,266,001)

Four mRNAs encoding novel salt-responsive proteins were selected for analysis. We compared the mRNA levels with the 2-DE data, and determined that all of the qRT-PCR results were in good agreement with the2-DE data (Fig. 5).

### Immunoblot analysis for RuBisCO activase and heat shock protein 70

**Fig. 6:**
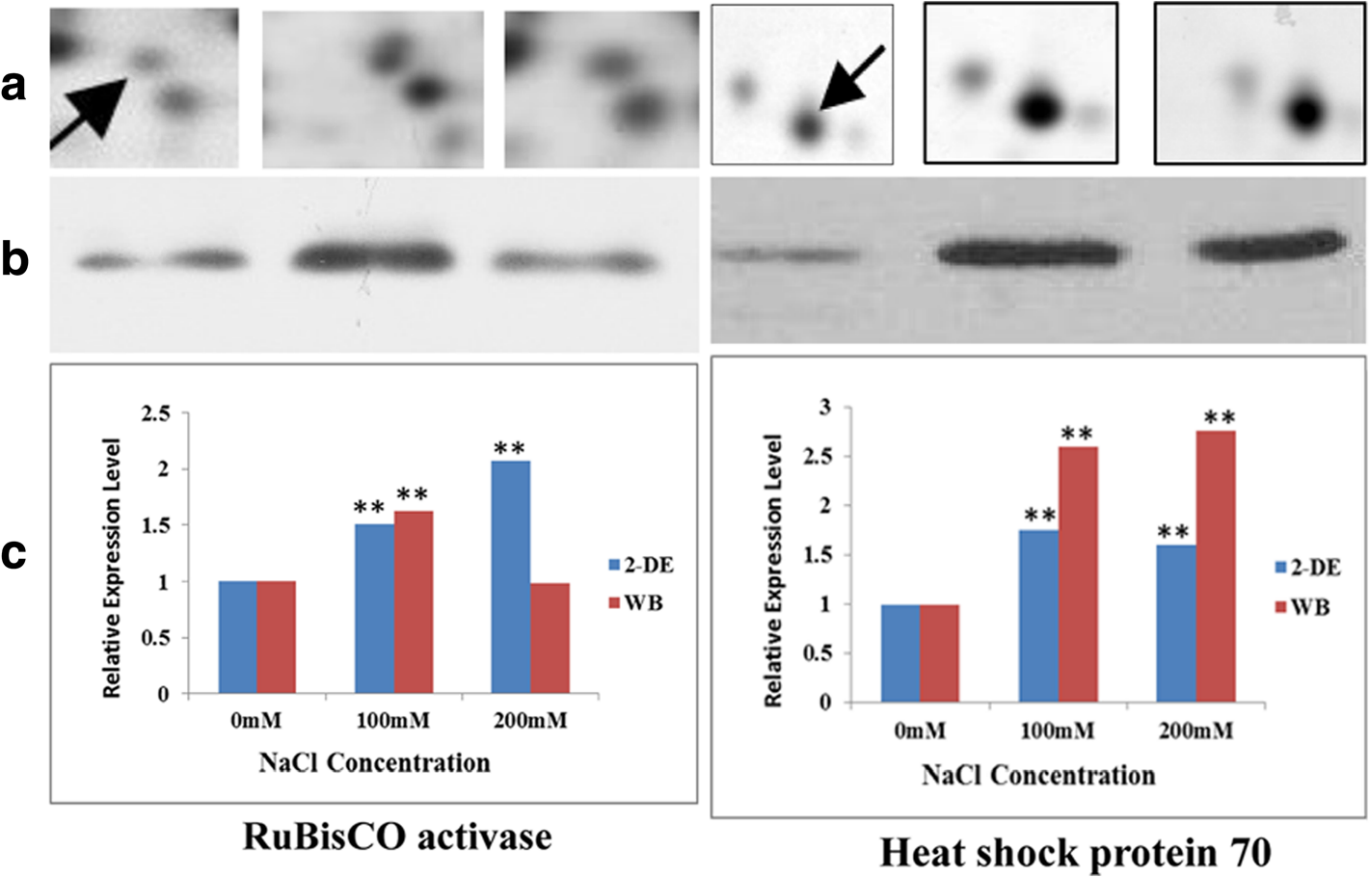
Western blot and 2-DE analysis of RuBisCO activase and HSP70 abundant patterns and the relative adundance level in alfalfa. **a** Antibodies against RuBisCO activase and HSP70 were used to detect the change of protein levels in alfalfa in response to salt stress treatment of the plants; **b** Image of RuBisCO activase and HSP70 spots on 2-DE gels; **c** Gray analysis of the relative adundance of RuBisCO activase and HSP70 (spots 8 and R1) were compared with the 2-DE data. Significant differences were determined relative to each treatment using a student’s t-test [*P*-values <0.05 (*) and <0.01 (**)]

In the current study, the accuracy of 2-DE analysis was further validated by immunoblot analysis. Proteins of alfalfa roots and shoots were separated by one-dimensional SDS-PAGE, and immunoblot analysis was performed for Heat shock protein 70 and RuBisCO activase (Fig. 6).

In agreement with the changes in protein abundance observed by 2-DE, Heat shock protein 70 showed an increased amount in response to 100 mM and 200 mM NaCl treatment. RuBisCO activase immunoblot analysis revealed an increase amount in response to 100 mM NaCl treatment, while the 200 mM value is not significantly different from control. This result is different in 2-DE analysis.

## Discussion

Salt stress decreased the growth of both shoots and roots, and this is a well-known physiological change in alfalfa. However, the mechanisms that regulate salt adaptation in alfalfa are complicated and are not well understood. In this study, through a combination of biochemical and proteomic approaches, we were able to undertake a comprehensive analysis of salt stress responses and defense in alfalfa shoots and roots for the first time.

### Proteins involved in photosynthesis

Photosynthesis is one of the most important processes to be affected by salinity. The effects of salt stresses on photosynthesis are either direct, such as diffusion limitations through the stomata and the mesophyll, and alterations in photosynthetic metabolism, or secondary, such as the oxidative stress arising from the superimposition of multiple stresses [19]. Therefore, it was not surprising to observe that the abundance of eight proteins involved in photosynthesis were altered under NaCl treatment. Among these proteins, three thylakoid membrane proteins: cytochrome b6-f complex iron-sulfur (Cyt b6/f, spot 12), chlorophyll a/b binding protein (CAB, spot 16), and chloroplast oxygen-evolving enhancer protein 1 (OEE1, spot 26) were down-regulated by salt-stress. These proteins are involved in the light reactions, including electron transfer, light-harvesting, and light-induced oxidation of water. As previously pointed out, salt stress can limit CO_2_ fixation, and the reducing power production rate is greater than the rate of its use by the Calvin cycle. The excess reducing power will induce the production of reactive oxygen species, thus the protection mechanisms against excess reducing power are an important strategy for combating salt stress [19]. In our study, the down-regulated proteins involved in the light reactions will help alfalfa to reduce reducing power production. However, in some salt-tolerant plants, such as *Thellungiella halophila*, *Agrostis stolonifera*, and *Kandelia candel*, salt stress induced the up-regulation of light reaction proteins [20–22].

Three spots (spots 2, 3, and 4) were identified as RuBisCO subunits. RuBisCO, created either through the carboxylation or oxygenation of ribulose-1,5-bisphosphate with carbon dioxide or oxygen, respectively, is composed of eight large subunits and eight small subunits. RuBisCOs are the most common enzymes in plants and salt stress induced altered abundance of RuBisCO subunits have been found in almost all green plant leaves. Previous studies showed that oxidative stress may lead to small-subunit degradation, which subsequently leads to translational arrest of the large subunit. Alternatively, oxidative stress could initially arrest large subunit translation, resulting in a rapid degradation of the unassembled small subunits [23]. It is noteworthy that a RuBisCO activase protein (spot 8) was up-regulated as the salt concentration rose. The principal role of RuBisCO activase is to release inhibitory sugar phosphates, such as ribulose-1,5-biphosphate, from the active RuBisCO sites so that CO_2_ can activate the enzyme controlling carbamylation. Therefore it ultimately determines the proportion of available RuBisCO active sites that are catalytically competent [24, 25]. Salt-stress directed reduction in stomatal conductance and subsequent low CO_2_ levels, together with the up-regulation of activase activity, may be required in order to induce salt stress tolerance. Previous studies have shown that salt stress induced the up-regulation of RuBisCO activase in rice leaf lamina, barley, and wild halophytic rice [26–28].

A CP12 (spot 6) protein was down- regulated after the 200 mM NaCl treatment in the shoots. CP12 is a small nuclear encoded chloroplast protein, which, in chloroplasts, is oligomerized with phosphoribulokinase (PRK) and NADP^+^-GAPDH in the presence of NAD(H) to generate a PRK/CP12/GAPDH complex. However, the complex dissociates in the presence of NADP(H). In Synechococcus, the oligomerization of CP12 with PRK and GAPDH regulates the activities of both enzymes and thus the carbon flow from the Calvin cycle to the oxidative pentose phosphate cycle [29]. In this manner, the down-regulated of CP12 seem to induce by the depression of photosynthesis.

### Stress responsive proteins form the largest protein group

Salt stress causes the production of excessive reactive oxygen species (ROS), which oxidize cellular components and irreversibly damage plant cells. In the present study, a total of 8 identified proteins were found to obviously relate to anti-oxidative reactions in alfalfa seedling roots and shoots in response to salt stress. All these proteins were up-regulated under 100 mM and /or 200 mM NaCl stress in alfalfa shoots and/or roots. The 8 proteins included 4 ascorbate peroxidases (spots 23, R16, R21, R23), 2 glutathione peroxidase (spots 25, R4), 1 ferritin protein (spot R18), and 1 quinone reductase family protein (spot 19). These proteins are major ROS-scavenging proteins, providing plant cells with highly efficient machinery for detoxifying H_2_O_2_ and the other ROS. However, all of these identified proteins had more distribution in root than in shoots. Our proteomics results might indicate that alfalfa seedling would increase ROS-scavenging proteins in response to salt stress and root may have stronger ROS-scavenging capability than shoot. REL and MAD are important indicators of membrane damage caused by ROS stress. In this study, the H_2_O_2_, REL and MAD contents were similar for all the NaCl concentrations, and all three were higher in the shoots than in the roots. A possible reason is that roots may have a better antioxidative defense system than the shoots. To validate the hypothesis, four enzymes involved in ROS scavenging were selected for activity analysis. It is important to note that the roots had higher SOD, APX and POD activities, and the shoots had higher CAT activities. CAT is known to have a lower affinity to H_2_O_2_ than POD (mM and μM range, respectively), and lower CAT activities were correlated to salt tolerance simply because large increases in CAT activity was not essential as long as POD and APX imposed tight controls on the H_2_O_2_ concentration [30]. This suggests that different mechanisms control the response to ROS.

In addition to the redox related proteins, plants have developed cross-tolerance mechanisms to be able to cope with different stresses. Some biotic stress-related proteins were induced under salt stress conditions, such as Pathogenesis-related protein 5(PR5, spot R9), Pathogenesis-related protein 2(PR2, spots 20, R27). PR2 is encoded by β-1,3-glucanase gene, and plant β-1, 3-glucanases are induced not only by pathogen infection, but also by other factors. Stress factors like wounding, drought, exposure to heavy metals, air pollutant ozone, and ultraviolet radiation can stimulate synthesis of β-1,3-glucanases in some plants [31]. However, there was few reported about it induced by salt stress. Previously, PR5 was gradually increased in abundance with increasing concentrations of NaCl in *Arabidopsis*, but the change was the opposite in *Thellungiella* [21]. PR proteins have been found to be induced in several plant species when they are infected by viruses, viroids, fungi or bacteria. In our study, the PR protein was induced by salt stress, which suggested that it had a special role in plant adaptation to salt stress, but whether it can be used as a potential salt stress marker in alfalfa needs further research. Moreover, some abiotic stress-related proteins, such as alcohol dehydrogenase (ADH, spots R10, R26) and harvest-induced protein (HI, spot 13) also respond to salt stress. ADH enzymes were traditionally of interest because of their activity during oxygen deprivation [32]. However, more recently, ADH gene expression and ADH activity have been shown to be affected by a number of other stresses [33, 34]. ADH1 has been found to be up-regulated in *Porteresia coarctata* under high salinity and this study also suggested that ADH1 was up-regulated when alfalfa was subjected to salt stress. HI proteins are involved in defense responses and the response to biotic stimulus, but their molecular details are poorly understood.

### The main energy metabolism associated proteins were down regulated

Salt-stress led to a reduction in photosynthesis, and thus to decreased carbohydrate synthesis. It also inhibited energy production. Energy production declined as the NaCl concentration increased. There were three proteins involved in glycolysis and the citrate cycle decreased after NaCl treatment.

The abundance of two glyceraldehyde-3-phosphate dehydrogenases (GAPDH, spots 18, R34) were altered in both the roots and shoots. Previous studies have shown that salt stress induces the up-regulation of GAPDH in rice leaves, OSRK1 transgenic rice roots, sugarcane, and in *Arabidopsis thaliana* roots [15, 35, 36]. The up-regulation of GAPDH may increase soluble sugars accumulation and provide more energy for plants under stress. It is therefore an indicator of stress tolerance. In this study, GAPDH was down-regulated in the shoots under 200 mM NaCl treatment, but was up-regulated in the roots under the 100 mM NaCl treatment. In addition, the abundance of a cytosolic phosphoglycerate kinase (cPGK, spot R24) was also altered in the roots. PGK is the seventh enzyme in the cycle that catalyzes the reaction of 1,3-biphosphoglycerate and ADP to produce 3-phosphoglycerate and ATP. GAPDH and PGK are crucial enzymes in the glycolysis cycle and showed the same abundance trends as GAPDH in the roots, which presumably reflects altered carbon flux patterns in response to the increased need for osmotic adjustment in the roots. Furthermore, a malate dehydrogenase mitochondrial (miMD, spot R7) was down-regulated under salt-stress in the roots. Overexpression of malate dehydrogenase in transgenic alfalfa enhances organic acid synthesis and confers tolerance to aluminum [37]. Malate dehydrogenase (cytoplasmic) was up-regulated under NaCl stress in cucumber roots and young rice panicles [38, 39], whereas in our study, malate dehydrogenase was down-regulated under salt stress. A possible reason is that the MD cellular localization was different in each species.

Up abundance of the ATP β synthase subunit was observed in both the roots and shoots under salt stress (spots 11, R14). ATP synthase includes two regions: an F0 region and F1 region consisting of α, β, γ, δ, and ε subunits. ATP synthase β subunit induction by salt stress has been reported in plants [12, 40, 41] and those studies show a positive correlation between the abundance of ATP synthase and a plant’s ability to resist salt stress. A nucleoside diphosphate kinase 1 (NDPK1, spot R12) was down-regulated under salt stress NDPKs are housekeeping enzymes, and their main function is to transfer a γ-phosphate from ATP to a cognate nucleoside diphosphate, thereby balancing the nucleoside pool. NDPK has been reported in response to drought [42, 43], cold [44], high temperature [45], and salt stresses [22, 38]. A recent study showed that NDPK 2 was involved in salt stress and H_2_O_2_ signaling in *Arabidopsis thaliana* [46].

### Salt-stress induced some secondary metabolism proteins

Secondary metabolism is a unique plant characteristic, is critical growth and development, and also allows plants to adapt to changing environments. Plant cells produce a vast number of secondary products, and some compounds are restricted to single species.

Flavonoids are ubiquitous plant secondary products that are best known as the characteristic red, blue, and purple anthocyanin pigments seen in plant tissues. In our study, isoflavone reductase (IFR, spot R19), isoliquiritigenin 2′-O-methyltransferase (IOMT, spot R5), chalcone reductase (CHR, spot R13), and chalcone isomerase (CHI, spot R33) showed up-regulated by salt stress in the seedling roots under 100 mM. Previously, it has been reported that flavonoids act as attractants to pollinators and symbionts, as sunscreens to protect against UV irradiation, as allelochemicals, as antimicrobial and antiherbivory factors, and are involved in resistance to aluminum toxicity [47, 48]. It is noteworthy that the key enzyme involved in flavonoid metabolite production showed an up-regulation under moderate NaCl treatment, which suggested that flavonoids also respond to the salt stress.

It well known that plants accumulate compatible osmolytes and osmoprotectants that help them to resist salt and drought stress. A L-myo-inositol 1-phosphate synthase (MIPS, spot 17) was up-regulated by salt stress in the shoots. The structure of this protein has been well-studied and was found to be inherently salt-tolerant [49]. Previous studies have suggested that salt stress induced the accumulation of MIPS in *Mesembryanthemum crystallinum* and that it was slightly upregulated in *P. coarctata* [27, 50].

It has been reported that an number of amino acids increase in alfalfa following NaCl treatment [51]. Glutamine synthetase 58 (GS58, spot R2) was up-regulated by both the salt stress treatments. GS catalyzes the ATP-dependent condensation of ammonium with glutamate to yield glutamine, which then provides nitrogen groups for the biosynthesis of all nitrogenous compounds in the plant [52]. Because glutamate is a precursor of proline, GS activation may contribute to proline synthesis under salt stress [53]. Previous reports indicated that GS was up-regulated under salt stress in rice and *Arabidopsis* roots [15, 54, 55]. A 3-isopropylmalate dehydrogenase (IMD, spot R11), which is involved in Leu biosynthesis, and cobalamine-independent methionine synthase, decreased in abundance following 100 mM NaCl treatment, but were up-regulated following 200 mM NaCl treatment. In *Arabidopsis* roots, IMD decreased in abundance following NaCl treatment [15], and the abundances of two IMDs were also influenced by NaCl in *Oryza sativa* roots [12].

This study also revealed that many protein related hormones were synthesized in response to salt treatment in alfalfa. An allene oxide cyclase 2(AOC2, spot 1) protein was up-regulated under salt stress. AOC catalyzes the stereospecific cyclization of an unstable allene oxide to 9(S),13(S)-12-oxo-10,15(Z)-phytodienoic acid, the precursor of jasmonic acid (JA) [56]. JA is involved in a wide range of stress, defense, and developmental processes [57].Transgenic plants expressing a tomato allene oxide cyclase (AOC) also displayed enhanced salt tolerance [58]. Up-regulation of AOC2 protein has been previously reported in *Arabidopsis* under salt-stress [15, 21].Our results provide additional evidence that AOC improves plants survival under salt stress.

### Salt stress induced protein metabolism

Several proteins, involved in protein translation, processing and degradation, were identified. In our study, ribosomal proteins S4(RP S4, spot 10) was down-regulated under 200 mM NaCl in the shoots, while, RP L32 (spot R32) was up-regulated under salt stress in root. Ribosomes are essential ribonucleoprotein complexes that are engaged in translation and thus play an important role in metabolism, cell division, and growth. The levels of some of the ribosomal proteins decreased while some specific ribosomal components increased under salt stress were also reported on *Arabidopsis* [59] and *Gossypium hirsutum* [60]. Moreover, our data showed a eukaryotic translation initiation factor, 5A-2(eIF 5A-2, spot R28), was up-regulated under 100 mM NaCl, but down-regulated under 200 mM NaCl. EIF 5A-2 is part of the start site selection for the eIF2-GTP-tRNAi ternary complex within the ribosomal-bound preinitiation complex, and also stabilizes the binding of GDP to eIF2. Alter abundance of eIF5A protein has also been reported in rice leaf lamina and *SnRK2* transgenic rice under salt stress [26, 55]. Other eukaryotic translation initiation factor, such as eIF3I, were also found down-regualted under salt-stress in *Arabidopsis* roots and *Gossypium hirsutum* roots [15, 60]. All of these studies suggest that complicated regulation mechanisms may govern protein synthesis in order to help plants cope with salt stress.

Several proteins that promote the proper folding of proteins and/or prevent the aggregation of nascent or damaged proteins were detected. A protein, disulfide-isomerase A6 (PDI A6, spot R25), was up-regulated under 100 mM NaCl treatment but down-regulated under 200 mM NaCl treatment. A major function of PDI is as a chaperone, where it helps wrongly folded proteins to reach a correctly folded state without the aid of enzymatic disulfide shuffling [61]. Moreover, increased abundance of PDI protein has also been reported in rice roots [62] and *Gossypium hirsutum* roots [60]. A heat shock protein, 70 (HSP70, spot R1), was up-regulated under NaCl stress. HSPs are grouped into five families: HSP100s, HSP90s, HSP70s, HSP60s, and sHSPs (small HSPs), and may prevent misfolding and promote the refolding and proper assembly of the unfolded polypeptides generated. Experiments in which chrysanthemum HSP70 gene was overexpressed in *Arabidopsis thaliana* showed that an increase in HSP70 abundance led to a remarkable tolerance to heat, drought and salt [63]. In our study, HSP70 was up-regulated by exposure to high salinity, which suggested that the proteins play a crucial role in aiding the folding and assembly of proteins under salt stress in alfalfa seedlings. These results are similar to the results reported for *Kandelia candel*, *Saccharum spp*., *Brachypodium distachyon*, and *Oryza sativa* under salt stress [12, 20, 40, 64]. A proteasome subunit alpha type-2-B (spot R30), which is involved in protein degradation, accumulated under salt stress in the roots. The proteasome is a very large protein complex (26S) containing a 20S core particle, and is a multicatalytic protease that degrades proteins using an ATP-dependent mechanism by which cells regulate the concentration of particular proteins and degrade misfolded proteins [65]. The degradation process yields peptides that are about seven to eight amino acids long, which can then be further degraded into shorter amino acid sequences that can be used to synthesize new proteins [66]. In *Brachypodium distachyon*, a proteasome subunit was down-regulated in the salt-treated group but up-regulated in the recovery group, which suggested that it was mainly involved in abnormal condition recovery rather than in the defense against salt stress [64]. In our study, the refold-associated proteins were up-regulated, which suggested that alfalfa handles misfolded proteins mainly through refolding. One possible reason is that energy production is depressed under salt stress and degradation is an energy-consuming process.

### Transcriptional and translational control is a part alfalfa’s response to salt stress

Under salt stress, many response and defense-related genes are stimulated by upstream transcription regulatory factors, but the genes involved in normal plant growth and development are inhibited [64]. Gene expression regulation is achieved at several levels, i.e. transcriptional, post-transcriptional, translational, and post-translational levels. For example, a maturase protein (spot 5) involved in post-transcription was down-regulated under 200 mM NaCl in the shoots. In vivo, most plant group II introns do not self-splice, but require the assistance of proteinaceous splicing factors, known as maturases [67, 68]. Maturase genes can be found in fungal and plant mitochondria, as well as in plant chloroplasts, and the down-regulation of this protein may be related to the translation of related genes. Two nucleic acid binding proteins (NABP1, spots 15, R35) showed altered abundance in both the shoots and roots under salt stress. NABP is a small and highly conserved protein with nucleic acid chaperone activity that binds single-stranded nucleic acids [69]. One group of NABPs is the cold shock domain (CSD) containing proteins, and these CSDPs are involved in various cellular processes, including adaptation to low temperatures, cellular growth, nutrient stress, and the stationary phase [70, 71]. RNA-binding proteins (RBPs) have crucial roles in various cellular processes, such as cellular function, transport, and localization. They also play a major role in post- transcriptional control of RNAs, such as splicing, polyadenylation, mRNA stabilization, mRNA localization, and translation. In this study, three RNA binding proteins (spots 7, 24, R22) were identified.

### Salt stress depressed the abundance of proteins involved in cellular processes

Salt stress decreased the growth of alfalfa, and several proteins associated with the dynamic changes of cellular processes were found in the current study. The actin cytoskeleton plays a critical role in plant development by regulating a number of fundamental cellular processes, including cell division, cell expansion, organelle motility, and vesicle trafficking [72, 73]. The dynamic reorganization of actin is modulated by the specific activity of a number of actin binding proteins (ABPs) that either promote or inhibit actin polymerization. Actin-depolymerizing factor (ADF) is one of the most highly expressed ABPs in plants that modulate the dynamic organization of the actin cytoskeleton by promoting filamentous actin disassembly [74]. ADFs were induced by salt stress, drought, and cold in cereal plants [43, 54, 75], which suggested that ADFs might be required for osmoregulation under osmotic stress. According to Yan [4], increased ADF levels under salt stress may result in depolymerization of actin filaments and enhanced K^+^ influx through the inward rectification of potassium channels, which helps to restore ion homeostasis. In this study, two spots, down-regulated under 200 mM salt stress in the roots, were identified as ADF (spots R6, R31). Another important ABP is profilin(spot R8), which was also first up and then down-regulated as the salt concentrations rose in the roots. These differences may be due to the need for growth under salt stress.

Two cell division cycle proteins (CDC48, spots 21, 22) were up-regulated under 100 mM NaCl. CDC48 belongs to the ATPases associated with proteins and has many cellular activities. They are believed to function as chaperones and to regulate cell-cycle progression, membrane fusion, and the destruction misfolded secretory proteins [76, 77]. Recent studies have shown that virus movement is impaired by the overexpression of CDC48, suggesting that CDC48 controls virus movement by removal of movement proteins from the endoplasmic reticulum-transport pathway and by interfering with protein movement using microtubule dynamics [78].

### Signal transduction and ion transport

Salt stress is first perceived by putative sensors in the root cell membranes and these signals are transmitted to the cellular machinery to regulate gene expression and changes in cellular metabolism designed to prevent or minimize the deleterious effects of abiotic stress. This signaling is mediated by different kinds of secondary messengers, such as Ca^2+^. Salinity induced increases in cytoplasmic free calcium ([Ca^2+^]_cyt_) and fluctuations in [Ca^2+^]_cyt_ provide a means for relatively rapid responses and may lead to specific changes in gene expression programs [79]. Two annexin proteins (spots R15, R17) were identified in our study. Annexins are a multigene, multifunctional family of Ca^2+^-dependent membrane-binding proteins found in both animal and plant cells, and certain annexins may be targets of [Ca^2+^]_cyt_ fluctuations [80]. In *Arabidopsis thaliana*, annexins have been shown to mediate osmotic stress and abscisic acid signal transduction [81]. In alfalfa, annexin is up-regulated in response to osmotic stress, abscisic acid (ABA), and drought [82].

A plasma membrane H^+^-ATPase (PM H^+^-ATPase, spot R3) was also up-regulated under salt stress. A response to the accumulation of Na^+^ ions in the cytosol is their compartmentalization within the vacuole, while another response is to extrude them from the cell. In each case, the active Na^+^ efflux requires a H^+^ gradient across the vacuolar membrane enerated by stimulating protein expression of the vacuolar H ^+^-ATPase [83].The accumulation of PM H^+^-ATPase gene mRNA was induced by NaCl and this has been found to occur in glycophytes and halophytes. In rice, a salt-tolerant mutant highly expressed PM H^+^-ATPase in its roots, compared to the non-mutant variety [84]. Therefore, the increased abundanceof plant plasma membrane H^+^-ATPase many play an important role in salt stress tolerance in alfalfa.

## Conclusion

In summary, we found significant physiological and protein abundance differences during salt treatment in alfalfa. Quantitative analysis of more than 850 spots on 2D gels showed significant variations in of 36 protein spots from the roots and 27 protein spots from the shoots, which were confidently identified by MS/MS. These DAPs are mainly involved in the biological processes of photosynthesis, stress and defense, carbohydrate and energy metabolism, second metabolism, protein metabolism, transcriptional regulation, cell wall and cytoskeleton metabolism, membrane and transport, signal transduction. The diverse array of proteins affected by salt stress conditions indicates that there is a remarkable flexibility in alfalfa roots and shoots metabolism, which may contribute to its survival in salinity conditions. Further analysis demonstrated that the primary mechanisms underlying the ability of alfalfa seedlings to tolerate salt stress were photosynthesis, detoxifying and antioxidant, secondary metabolism, and ion transport. In parallel, physiological data, including REL, MAD and H_2_O_2_ contents, and the activities of antioxidant enzymes all correlated well with our proteomic results. It is worth emphasizing that some novel salt-responsive proteins were identified, such as CP12, pathogenesis-related family proteins, harvest-induced protein, isoflavone reductase, isoliquiritigenin 2′-O-methyltransferase, chalcone reductase, chalcone isomerase. qRT-PCR was used to study the gene expression levels of thefour above-mentioned proteins; four patterns are consistent with those of induced protein. These novel proteins provide a good starting point for further research into their functions using genetic or other approaches. These findings significantly improve the understanding of the molecular mechanisms involved in the tolerance of plants to salt stress.

## Abbreviations

2-DE: Two-dimensional gel electrophoresis
ABP: Actin binding proteins
ADF: Actin-depolymerizing factor
ADH: Alcohol dehydrogenase
AOC: Allene oxide cyclase
APX: Ascorbic acid peroxidase
CAB: Chlorophyll a/b binding protein
cAPX: cytosolic ascorbate peroxidase
CAT: Catalase
CDC: Cell division cycle proteins
CHI: Chalcone isomerase
CHR: Chalcone reductase
cPGK: cytosolic phosphoglycerate kinase
ctbAPX: chloroplast thylakoid-bound ascorbate peroxidase
Cyt b6/f: Cytochrome b6-f complex iron-sulfur
DAPs: Differentially abundant proteins
eIF: Eukaryotic translation initiation factor
GAPDH: Glyceraldehyde-3-phosphate dehydrogenases
GO: Gene ontology
GPX: Glutathione peroxidase
GS: Glutamine synthetase
HI: Harvest-induced protein
HSP: Heat shock protein
IFR: Isoflavone reductase
IMD: 3-Isopropylmalate dehydrogenase
IOMT: Isoliquiritigenin 2′-O-methyltransferase
MD: Malate dehydrogenase
MDA: Methane Dicarboxylic Aldehyde
MIPS: L-myo-inositol 1-phosphate synthase
NABP: Nucleic acid binding proteins
NDPK1: Nucleoside diphosphate kinase 1
OEE1: Chloroplast oxygen-evolving enhancer protein 1
PDI: Protein disulfide-isomerase
POD: Peroxidase
PR: Pathogenesis-related
RBP: RNA-binding proteins
REL: Electrolyte leakage analyses
ROS: Reactive oxygen species
RP: Ribosomal proteins
SOD: Superoxide dismutase
